# Thoracoscopic resection of bilateral multiple superior mediastinal neurofibromas

**DOI:** 10.1186/s13019-021-01690-w

**Published:** 2021-10-20

**Authors:** Yoko Azuma, Naobumi Tochigi, Atsushi Sano, Takashi Sakai, Akira Iyoda

**Affiliations:** 1 Division of Chest Surgery, Department of Surgery, Toho University School of Medicine, 6-11-1, Omori-nishi, Ota-ku, Tokyo, 143-8541 Japan; 2 Department of Surgical Pathology, Toho University School of Medicine, 6-11-1, Omori-Nishi, Ota-ku, Tokyo, 143-8541 Japan

**Keywords:** Bilateral multiple neurofibromas, VATS, Superior mediastinum tumors, Neurofibromatosis

## Abstract

**Background:**

The indications for surgical resection concerning multiple bilateral neurofibromas in the superior mediastinum remain controversial, because vascular injury or development of postoperative Horne syndrome are concerned.

**Case presentation:**

A 60-year-old woman presented with multiple nodules in her right neck and bilateral chest cavity tops which indicated neurofibromatosis. The thoracic masses grew slowly over 9 years, and she then underwent a 2-stage resection starting with the left to right side. Bilateral tumors were completely removed via video-assisted thoracic surgery. The patient’s postoperative course was uneventful, without postoperative Horner syndrome.

**Conclusions:**

To the best of our knowledge, this is the first case of multiple bilateral superior mediastinal neurofibromas resected from the pulmonary apices via thoracoscopy. We selected a minimally invasive pure video-assisted thoracoscopic surgery approach and enucleated some tumors to avoid nerve injury. This approach may be safe and useful for multiple neurofibromas in patients with neurofibromatosis.

## Background

Mediastinal neurogenic tumors usually arise from an intercostal nerve or the sympathetic chain, and they comprise the majority of posterior mediastinal tumors. Most of these tumors are benign, and associated with a good prognosis after complete surgical resection. However, the indications for surgical resection concerning multiple bilateral neurofibromas in the superior mediastinum remain controversial. Herein, to our best knowledge, we report the first case of multiple bilateral superior mediastinal neurofibromas completely resected via video-assisted thoracic surgery.

## Case presentation

A 60-year-old woman was found to have abnormal shadows near both pulmonary apices on routine chest radiography at a local hospital. Chest computed tomography (CT) showed multiple nodules in her right cervical neck and bilateral chest cavity tops. The findings were diagnosed as multiple neurogenic tumors, and the patient was placed under observation. The thoracic masses grew slowly over 9 years, and she was referred to our hospital for resection.

**Fig. 1:**
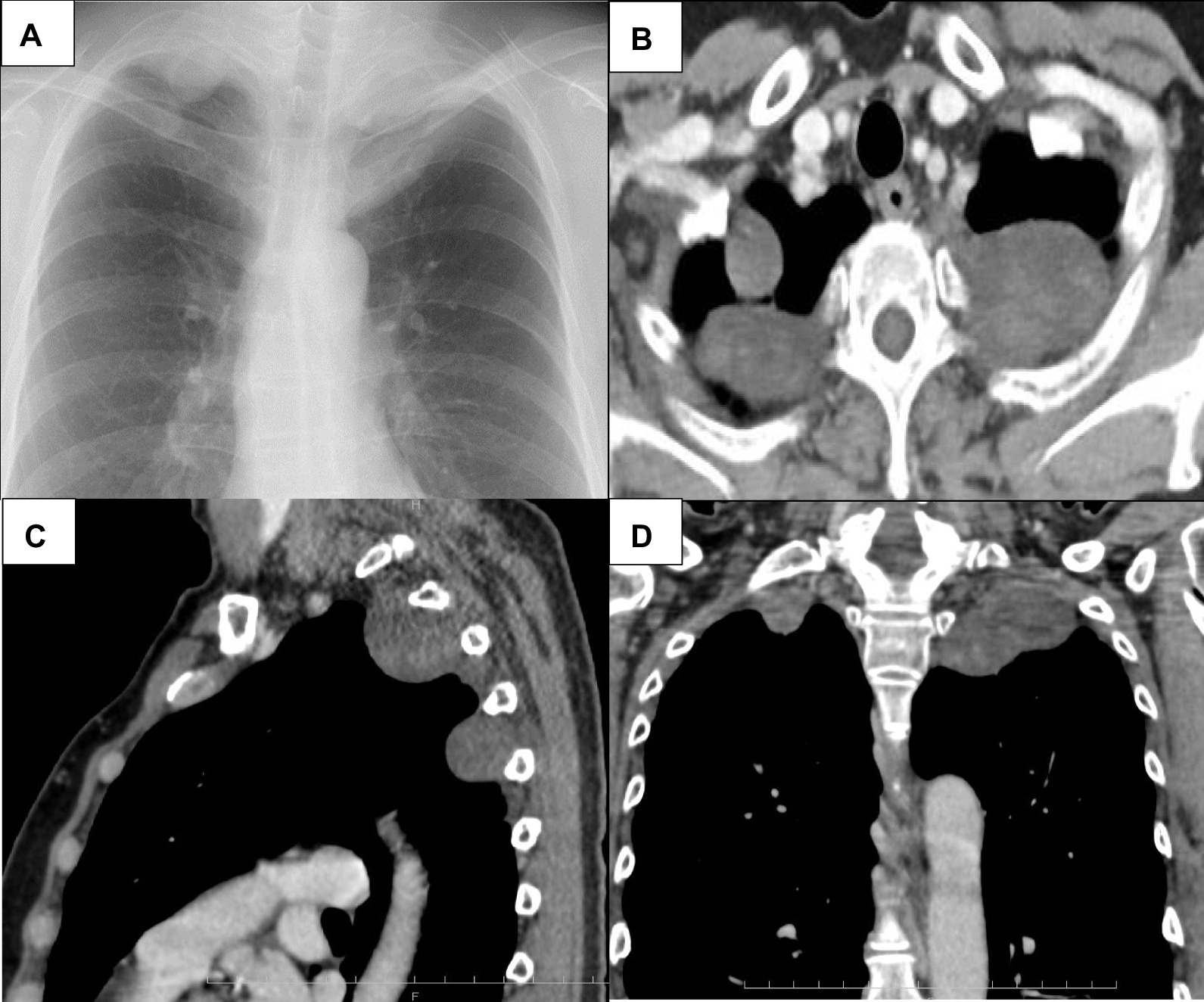
**A** Chest X-ray shows multiple shadows in both pulmonary apices. **B** Axial, **C** sagittal, and **D** coronal CT view shows well defined, multiple superior mediastinal masses located paravertebrally

**Fig. 2:**
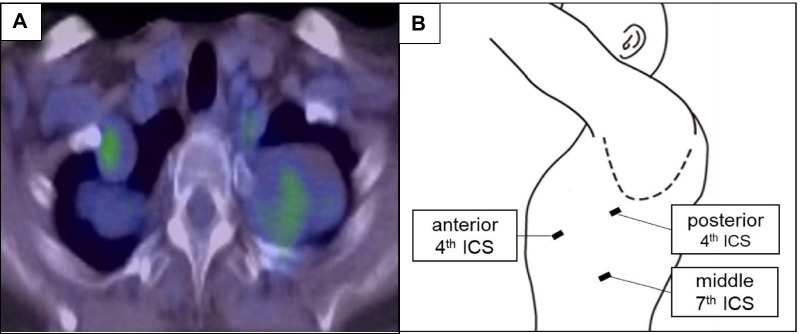
**A** Axial FDG-PET view shows elevated FDG uptake in the tumors. **B** Schema of surgical approach

The patient had no cutaneous lesions, and her family history was negative for neurofibromatosis. Chest radiography revealed abnormal shadows near both pulmonary apices (Fig. 1A). CT showed multiple well-defined masses of up to 3.5 cm in bilateral chest cavity tops. The thoracic masses were located in a row and contacted the 1st to 5th ribs and vertebral body (Fig. 1B–D). Magnetic resonance imaging revealed multiple well circumscribed T2-weighted hyperintense and T1-weghted isointense masses in her neck and bilateral chest cavity tops. F18-fluorodeoxyglucose positron emission tomography (FDG-PET) showed abnormal FDG uptake in the tumors, with a maximum standardized uptake value of 3.15 (Fig. 2A). These imaging findings suggested multiple neurogenic tumors. Because of worry about the malignant potential of the masses and expansion of the masses into intervertebral foramina, we proceeded with surgical resection of the thoracic masses for therapeutic diagnosis.

**Fig. 3:**
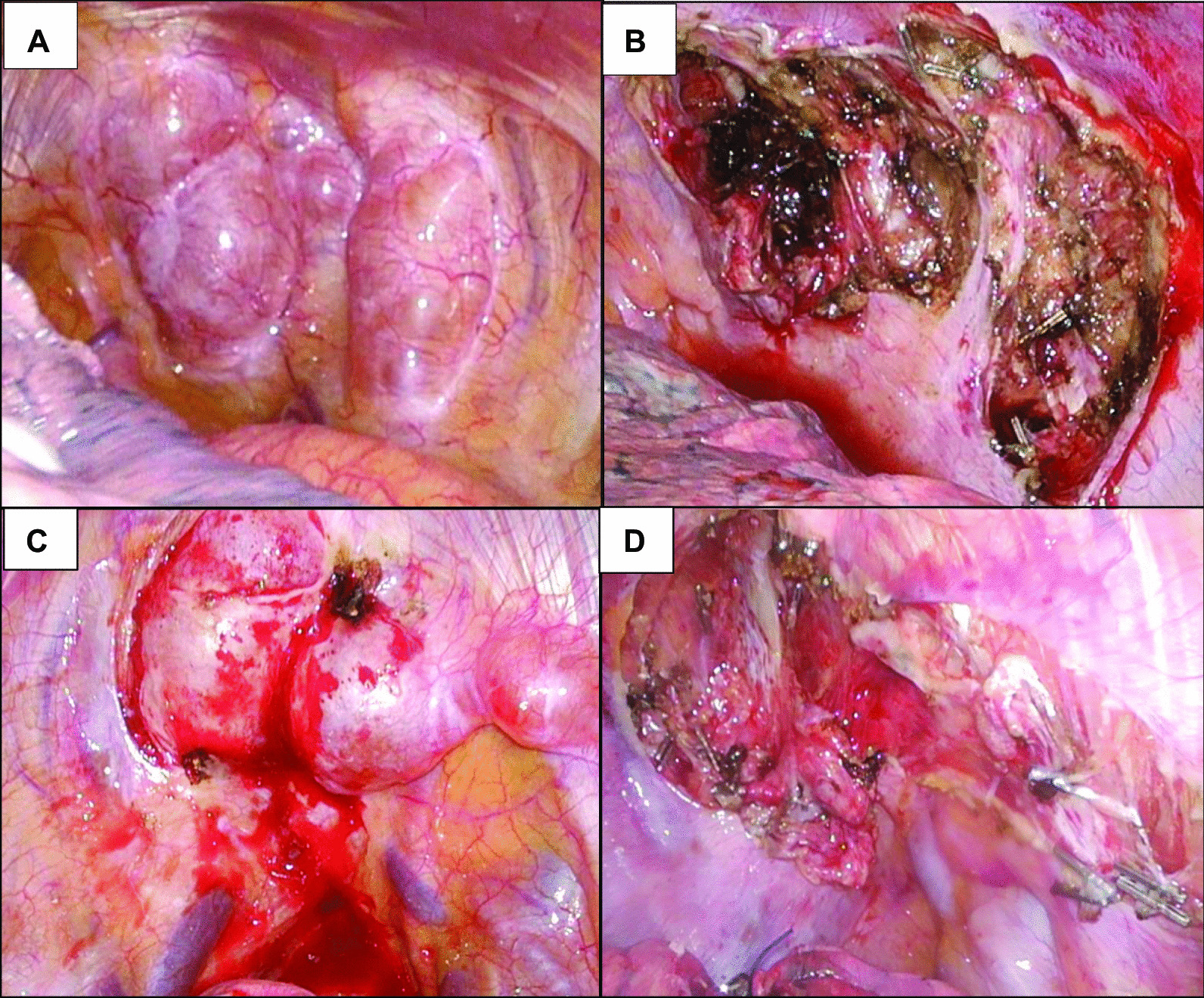
Intraoperative view. On the left side **A** tumors extending from the 1st to 5th intercostal spaces, and **B** were resected completely. On the right side **C** tumors extending from the 1st to 2th intercostal space, and **D** were resected completely

**Fig. 4:**
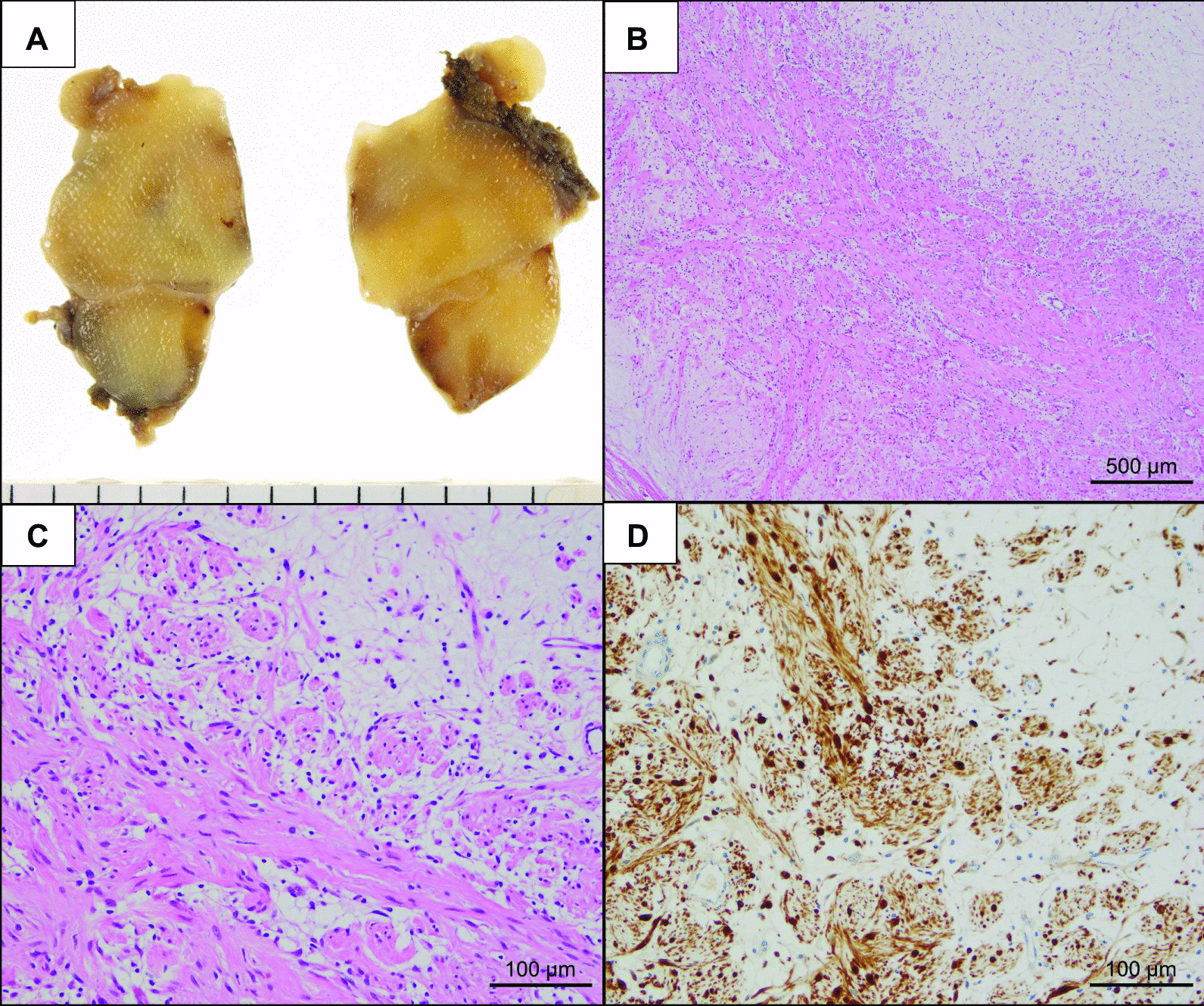
Histopathological findings of the resected tumors on the right side. **A** The cut surfaces of tumor specimens appear as a homogeneous yellowish-white. **B** The microscopic findings at magnification ×40 and **C** ×200. **D** Tumor cells were positive for S100 at magnification ×200

Since this case had multiple tumors and it was unclear whether safe and complete resection was possible, we decided to perform surgery from one side first. A two-stage resection was performed starting on the left, followed 8 months later by resection on the right to reduce the risk of the development of Horner syndrome after surgery. Bilateral tumors were removed completely via video-assisted thoracoscopic surgery (VATS). A 3-port approach was used as follows: incisions for 12-mm ports were made in the 7th intercostal space at the middle axillary line, and in the 4th intercostal space at the anterior and posterior axillary lines (Fig. 2B). On the patient’s left side there were 3 soft and well-defined capsuled tumors extending from the 1st to 5th intercostal spaces (Fig. 3A). One tumor arose from the sympathetic trunk and the others from intercostal nerves. The intercostal nerves associated with the tumors were dissected, and the sympathetic trunk was preserved by enucleating the tumor. The tumor capsule was thickened and the dissection was relatively easy. The masses were completely resected without involving the spinal cord (Fig. 3B). On the patient’s right side, there were 3 similar tumors arising from the intercostal nerves in the 1st to 2th intercostal space (Fig. 3C), and complete resection was obtained (Fig. 3D). The resected tumors had an elastic consistency, and the cut surface appeared as a homogeneous yellowish-white tissue (Fig. 4A). The histopathological diagnosis of the tumors was neurofibroma with spindle-shaped cells and narrow nuclei without atypia (Fig. 4B, C). Immunohistochemical analysis revealed S-100 protein expression by the tumor cells (Fig. 4D). The patient’s postoperative course was uneventful, without development of postoperative Horner syndrome. She has remained free of recurrence of thoracic lesions and symptoms for 3 years after the first surgery.

## Discussion

After schwannoma, neurofibroma is the second most frequent adult mediastinal neurogenic tumor (MNT). Neurofibromas occur as isolated neoplasms or as multiple thoracic manifestations of neurofibromatosis [1]. Approximately 30–45% of patients have neurofibromas that are associated with neurofibromatosis [1]. Neurofibromatosis is comprised of 3 distinct diseases, as follows: neurofibromatosis type 1 (NF1: Von Recklinghausen disease), neurofibromatosis type 2 (NF2), and schwannomatosis. NF1 occurs most frequently as multiple neurofibromas, and shows characteristic clinical findings such as café-au-lait macules. NF2 shows as nervous system tumors (schwannomas involving the vestibular nerve or other regions of the nervous system, meningiomas, ependymomas, astrocytomas, and neurofibromas), peripheral neuropathy, ophthalmological lesions, and cutaneous lesions [2]. Our patient might have NF2, although she did not have lesions involving the vestibular nerve or other regions of the nervous system. We plan to monitor the patient for development of other nervous system tumors, especially tumors associated with the acoustic nerve.

The indications for surgical resection concerning multiple bilateral neurofibromas in the superior mediastinum remain unclear. Usually, MNTs account for 20% of all adult mediastinal neoplasms. They arise from peripheral nerves or nerve sheaths, and are almost exclusively located in the posterior mediastinum^1^. Most patients with MNTs are asymptomatic, although they can manifest various symptoms such as chest pain, back pain, cough, and dyspnea. The majority of MNTs are benign, and the prognosis is excellent for patients undergoing complete surgical resection. However, approximately 10% of patients with MNTs show extensions of tumors into the vertebral canal, the so-called “dumbbell tumors” [3]. The surgical procedure for dumbbell tumors remains challenging and highly invasive procedure. Moreover, patients with neurofibromatosis are at increased risk for malignant transformation of their neurogenic tumors [1]. Because in our patient the tumors located paravertebrally could have expanded into the intervertebral foramina, we decided on performing surgery sooner rather than later.

Three previous surgical cases have been reported on multiple bilateral neurofibromas arising from the vagal nerve in the middle mediastinum [4–6]. The surgical approaches varied, and included thoracotomy, median sternotomy, and thoracoscopic surgery on 1 side only. In our case, tumors located in the thoracic apex near the stellate ganglion; therefore, we were concerned about vascular injury and the development of postoperative Horne syndrome. VATS is a minimally invasive approach that has been reported to be safe for the resection of intrathoracic neurogenic tumors. Especially, grasping the microanatomy by the VATS approach is useful for narrow and intricate spaces such as the thoracic apex. Endo et al. [7] suggested that tumor enucleation via VATS was preferable for apical well capsulated benign neurogenic tumors.

## Conclusions

We decided upon a minimally invasive pure VATS approach and enucleated some tumors to avoid nerve injury. This approach may be safe and useful for multiple MNTs in patients with neurofibromatosis.

## Data Availability

The data supporting the conclusions of this article are included within the article.

## Abbreviations

CT: Chest computed tomography
FDG-PET: F18-fluorodeoxyglucose positron emission tomography
VATS: Video-assisted thoracoscopic surgery
MNT: Mediastinal neurogenic tumor
NF: Neurofibromatosis
